# The High-Sensitivity C-Reactive Protein/Albumin Ratio Predicts Long-Term Oncologic Outcomes after Curative Resection for Hepatocellular Carcinoma

**DOI:** 10.3390/jcm7060139

**Published:** 2018-06-07

**Authors:** Tak Kyu Oh, Young-Rok Choi, Jai Young Cho, Yoo-Suk Yoon, Ho-Seong Han, In Sun Park, Jung-Hee Ryu

**Affiliations:** 1 Department of Anesthesiology and Pain Medicine, Seoul National University Bundang Hospital, Seongnam, 166 Gumi-ro, Bundang-gu, Seongnam 13620, Korea; airohtak@hotmail.com (T.K.O.); 54310@snubh.org (I.S.P.); 2 Department of Surgery, Seoul National University Bundang Hospital, Seongnam, 166 Gumi-ro, Bundang-gu, Seongnam 13620, Korea; choiyoungrok@gmail.com (Y.-R.C.); jychogs@gmail.com (J.Y.C.); yoonys@snubh.org (Y.-S.Y.); hanhs@snubh.org (H.-S.H.); 3 Department of Anesthesiology and Pain Medicine, Seoul National University College of Medicine, 103 Daehak-ro, Jongno-gu, Seoul 03080, Korea

**Keywords:** hepatobiliary-surg, prognostic factor, surgery

## Abstract

High-sensitivity C-reactive protein (hsCRP) is a prognostic factor for hepatocellular carcinoma (HCC), while albumin is known to be a disease severity index of the malnutrition status in HCC patients. The present study investigated the association between postoperative hsCRP/albumin ratio and both overall survival (OS) and recurrence-free survival (RFS) following HCC surgery. This retrospective observational study examined the medical records of 389 patients who underwent resection for HCC between 2004 and 2013. Postoperative day 0–1 hsCRP/albumin ratio was collected, and the optimal postoperative mortality cut-off point was derived using receiver operating characteristics (ROC) analysis. A postoperative hsCRP/albumin ratio increase of 1.0 was associated with a 1.171-fold increase in mortality (hazard ratio (HR): 1.171, 95% confidence interval (CI): 1.072–1.278, *p* < 0.001) and a 1.19-fold increase in recurrence (HR: 1.190, 95% CI: 1.108–1.278, *p* < 0.001). The hsCRP/albumin ratio cut-off point was found to be 0.625 and 0.500. When patients were grouped by this cut-off point, the >0.625 group showed a 2.257-fold increase in mortality (HR: 2.257, 95% CI: 1.470–3.466, *p* < 0.001), and the >0.500 group showed a 1.518-fold increase in recurrence (HR: 1.518, 95% CI: 1.125–2.050, *p* = 0.006).

## 1. Introduction

Liver cancer is the second leading cause of cancer-related mortality worldwide, causing 745,000 deaths per year [1]. Hepatocellular carcinoma (HCC) accounts for 70%–85% of primary liver cancers and typically has a poor prognosis [2]. Currently, the treatment of HCC mostly follows the options presented in published treatment guidelines [3,4] and multiple treatment options, including surgery, are utilized with the aim of complete cure [5,6]. Despite these multiple treatment options, the recurrence rate for HCC is approximately 70% [7], and subsequently, the overall survival (OS) and recurrence-free survival (RFS) of patients with HCC remains poor [8].

High-sensitivity C-reactive protein (hsCRP) is the most sensitive protein for the detection of inflammation and is also associated with the prognosis of a number of different cancers in terms of cell differentiation and proliferation [9,10]. Albumin is a protein that reflects a given patient’s nutritional state and is known to be associated with postoperative mortality [11,12]. Based on these relationships, high hsCRP and low albumin levels may be associated with increased patient mortality. Previous investigations have also reported that the hsCRP/albumin ratio is predictive of prognosis in patients admitted to the emergency room [13] and those with infection [14]. 

The hsCRP has recently been reported to reflect prognosis in liver cancer patients [15]. In previous studies that did not use hsCRP, CRP was investigated as a marker to predict survival and recurrence in patients with HCC [16,17]. However, hsCRP is known to have better sensitivity for inflammation even when CRP is within the normal range, with its effective use as a prognostic tool in early stage HCC being first reported in 2015 [18]. Around the same time, preoperative hsCRP was also reported to be useful in predicting long-term oncologic outcomes after surgical resection for HCC [19]. Nevertheless, these previous studies did not use the hsCRP/albumin ratio as a prognostic factor to predict long-term oncologic outcomes for HCC patients [18,19]. Given that the CRP/albumin ratio has already been reported to be a useful prognostic factor for long-term oncologic outcomes in patients newly diagnosed with HCC [20], the hsCRP/albumin ratio, which uses the more sensitive hsCRP over CRP, could be an important prognostic factor for HCC cases receiving surgery. Therefore, the aim of the present study was to investigate the effects of postoperative hsCRP/albumin ratio on OS and RFS in patients who have undergone curative resection for HCC.

## 2. Materials and Methods

### 2.1. Patients

This retrospective, observational study was approved by the Institutional Review Board (IRB) of Seoul National University Bundang Hospital (SNUBH) (approval number: B-1712-438-104, approval date: 4 December 2017). As this was a retrospective review of patient medical records, the requirement for informed consent was waived by the IRB. This study reviewed the medical records of all adult patients aged 20 years or older who were diagnosed with HCC and underwent curative resection at SNUBH during a 10-year period, from January 2004 to December 2013. The exclusion criteria were as follows: (1) incomplete medical record; (2) no hsCRP or albumin measurement on postoperative day (POD) 0 or 1; (3) follow-up loss. 

### 2.2. Surgical Management for Hepatocellular Carcinoma at SNUBH

HCC resections are routinely performed at SNUBH by an experienced hepatobiliary surgical team using either a laparoscopic technique or laparotomy [21]. The decision to perform laparoscopy was made by a multidisciplinary team based on the state of the tumor and the patient’s condition [22]. Anesthesia was administered by professional anesthetists using standardized techniques; usually, an inhalation agent (sevoflurane or desflurane) was used for anesthesia induction and maintenance. All medical staff used transfusion and vasopressors, as appropriate, to keep intraoperative hemodynamic variables stable. At SNUBH, hsCRP and albumin are routinely measured during the postoperative period as part of evaluating the patient’s postoperative condition. In addition, recurrence was evaluated in all patients after discharge through additional outpatient clinic-based follow-up.

### 2.3. Measurements and Outcomes

The following information was collected in this study: (1) patients’ preoperative demographic and clinical characteristics; (2) surgery-related information; (3) HCC-related pathologic information; (4) short-term (complications) and long-term oncologic outcomes (death or recurrence); (5) hsCRP (mg/L) and serum albumin (g/L) measured on POD 0 or 1. Surgeries that began as laparoscopy but involved intraoperative open conversion were considered to be laparotomies. The pathological staging of HCC was classified according to the American Joint Committee on Cancer 7th guidelines [23], and recurrence dates were based on the date that recurrence was diagnosed by radiologic findings. The hsCRP and albumin measurements used in this study were immediate postoperative laboratory test results, with the earliest measurement from POD 0–1 being used. In pathological staging, tumor size (cm) was defined as the maximal diameter of the largest HCC lesion. Combined resections included cases in which cholecystectomy, gastrectomy, or bowel resection was performed simultaneously with HCC resection.

The primary outcomes of this study were OS and RFS after surgery for HCC. The secondary outcome was OS and RFS according to the surgical technique used (laparoscopy vs. laparotomy). OS was defined as the number of postoperative days until death, and RFS was defined as the number of postoperative days until death or a diagnosis of recurrence.

### 2.4. Statistical Analysis

In order to determine the optimal cut-off value for the hsCRP/albumin ratio, a receiver operative characteristics (ROC) curve analysis was performed using the likelihood of mortality and recurrence after HCC resection. The cut-off point was defined as the point where sensitivity and specificity were equal. Univariate Cox regression analysis was used to examine the individual effect of each variable on OS. All variables with *p* < 0.1 in the univariate Cox regression model were then used in the final multivariate Cox proportional hazard model construction process. Here, two separate multivariate models were made: one in which subjects were divided into low- and high-hsCRP/albumin ratio groups, separated by the cut-off value, and one in which the hsCRP/albumin ratio was included as a continuous variable. The results of the multivariate Cox regression analysis are presented in terms of hazard ratios (HRs) and 95% confidence intervals (CIs). A log-minus-log plot was used to test whether each variable satisfied the Cox proportional hazard assumption.

To analyze the relationship between hsCRP and RFS, the same methods were used for OS were implemented. Subjects were again divided into laparoscopy and laparotomy groups for subgroup analysis, and a multivariate backward, stepwise Cox regression analysis was performed to investigate the effects of the hsCRP/albumin ratio on OS and RFS. In addition, Kaplan–Meier curves were used to determine OS and RFS after surgery for HCC in the high- and low-hsCRP/albumin ratio groups, classified according to the cut-off point derived above. The log-rank test was used to test the difference in mean OS and RFS between the subgroups. IBM SPSS version 24.0 software (IBM Corp., Armonk, NY, USA) was used for all statistical analyses, and results with *p* < 0.05 were considered statistically significant.

## 3. Results

We identified 418 patients who underwent curative resection for HCC at SNUBH between January 2004 and December 2013. Of these, 5 patients were excluded due to follow-up loss and 24 patients were excluded due to absent or inaccurate records for hsCRP or albumin on POD 0–1. There were 389 patients included in the final analysis, and their baseline characteristics are presented in Table 1. In the patients who underwent HCC surgery, the mean postoperative hsCRP/albumin ratio was 0.97 ± 1.46. When the subjects were divided by surgical technique, there were 232 patients who underwent laparotomy (59.6%) and 157 patients who underwent laparoscopy (40.4%). Among the patients who underwent surgery for HCC, 98 died (25.2%) during the postoperative follow-up period and 193 had recurrence (49.6%).

### 3.1. Cut-Off Value for hsCRP/Albumin Ratio

The ROC curve for hsCRP/albumin ratio and mortality after HCC surgery is shown in Supplementary Figure S1. The area under curve was 0.692 (95% CI: 0.635–0.748), and the hsCRP/albumin ratio cut-off point, where the sensitivity and specificity are equal, was 0.625. In addition, the ROC curve for the hsCRP/albumin ratio and recurrence after HCC surgery is shown in Supplementary Figure S2. The area under curve was 0.587 (95% CI: 0.536–0.637), and the hsCRP/albumin ratio cut-off point was 0.500.

### 3.2. Overall Survival and Recurrence-Free Survival after HCC Surgery

The results of the multivariate Cox regression analysis for mortality after HCC surgery are shown in Table 2. An increase of 1 in the postoperative hsCRP/albumin ratio was associated with a 1.171-fold increase in mortality risk (95% CI: 1.072–1.278, *p* < 0.001). Compared to the hsCRP/albumin ratio ≤0.625 group, the >0.625 group showed a 2.257-fold increase in mortality risk (95% CI: 1.470–3.466, *p* < 0.001). The results of the multivariate Cox regression analysis for recurrence after HCC surgery are shown in Table 3. An increase of 1 in the postoperative hsCRP/albumin ratio was associated with a 1.19-fold increase in recurrence risk (95% CI: 1.108–1.278, *p* < 0.001). Compared to the hsCRP/albumin ratio ≤0.500 group, the >0.500 group showed a 1.518-fold increase in recurrence risk (95% CI: 1.125–2.050, *p* = 0.006). 

**Figure 1:**
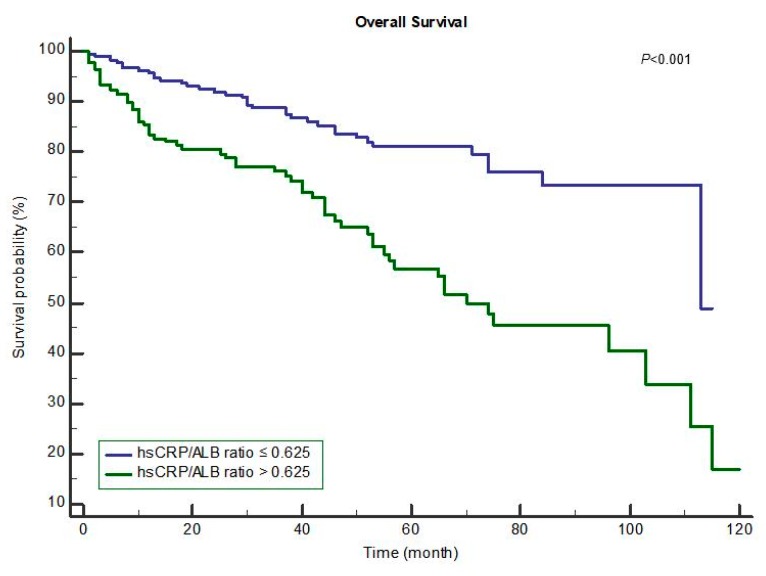
Overall survival after resection for hepatocellular carcinoma. Mean overall survival time ≤0.625: 95.10 months vs. >0.625: 70.94 months, *p* < 0.001.

**Figure 2:**
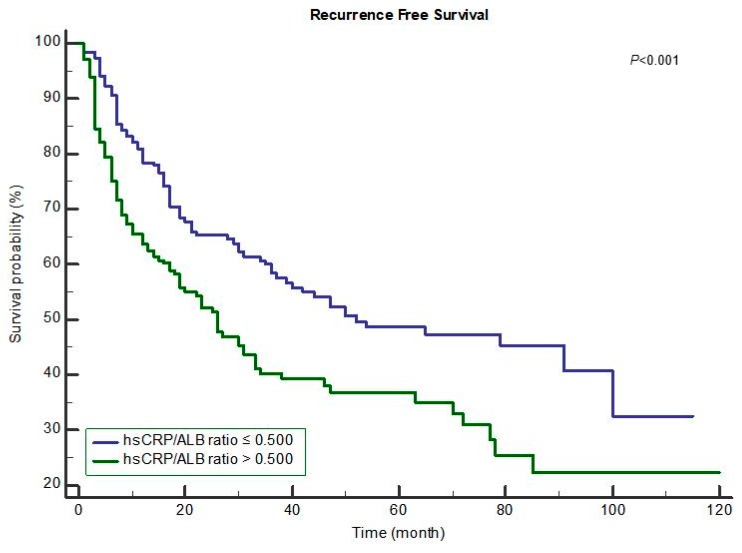
Recurrence-free survival after resection for hepatocellular carcinoma. Mean recurrence-free survival time ≤0.500: 62.64 months vs. >0.500: 46.63 months, *p* < 0.001.

The Kaplan–Meier curves for postoperative OS and RFS in the groups with hsCRP/albumin ratio ≤0.625 and >0.625 are shown in Figure 1 and Figure 2, respectively. Compared to the hsCRP/albumin ratio >0.625 group, the ≤0.625 group had a longer mean OS time (≤0.625 group: 95.10 months vs. >0.625 group: 70.94 months, *p* < 0.001; Figure 1). In addition, compared to the hsCRP/albumin ratio >0.500 group, the ≤0.500 group had a longer mean overall RFS time (≤0.500 group: 62.64 months vs. >0.500 group: 46.63 months, *p* < 0.001; Figure 2).

### 3.3. Subgroup Analysis: Laparoscopy versus Laparotomy for hsCRP/Albumin Ratio

Supplementary Table S1 shows the results of comparisons for characteristics between laparoscopy and laparotomy. The results of analyzing the effects of the hsCRP/albumin ratio on mortality and recurrence risk after HCC surgery in laparoscopy and laparotomy subgroups are shown in Table 4. With regards to mortality, in the hsCRP/albumin ratio >0.625 group, the laparotomy subgroup had a HR of 4.646 (95% CI: 2.082–10.366), and the laparoscopy subgroup had a HR of 4.581 (95% CI: 2.445–8.584). In addition, with regards to recurrence, in the hsCRP/albumin ratio >0.500 group, the laparotomy subgroup had a HR of 1.995 (95% CI: 1.307–3.045), and the laparoscopy subgroup had a HR of 1.483 (95% CI: 1.003–2.193).

## 4. Discussion

This is the first study to use postoperative hsCRP/albumin ratio to predict long-term oncologic outcomes of HCC patients who had undergone potentially curative resection. The results of the current study suggested that the postoperative hsCRP/albumin ratio is a prognostic marker to predict long-term oncologic outcomes after surgery for HCC. In addition, a cut-off point of 0.625 for mortality and 0.500 for recurrence was determined for the postoperative hsCRP/albumin ratio through an ROC analysis, and the risk of both recurrence and death also increased based on this cut-off point.

The present study used the postoperative hsCRP/albumin ratio instead of the preoperative value. Inflammatory markers, such as hsCRP, reflect the burden or progression of HCC tumor cells [24], but physical burdens from surgical procedures, poor general conditions, and combined resections can also increase the levels of inflammatory proteins during the immediate postoperative period [25]. Therefore, the hsCRP/albumin ratio used in our study not only reflects HCC status, but also the overall patient’s condition, which reflects physical burden or stress from surgical procedures, poor general conditions, and combined resections. For this reason, when compared with the conventional preoperative hsCRP assessment, postoperative hsCRP can be considered to have value in reflecting the extent of overall physical burdens from surgical procedures or patient condition. Moreover, whereas preoperative albumin reflects patient chronic comorbidity and malnutrition status [11,12], a drop in postoperative albumin is a marker of surgical stress and can be a predictor of clinical outcomes [26]. Therefore, the postoperative hsCRP/albumin ratio may be a prognostic scoring metric because it reflects overall physical burden from surgical procedures and patient condition.

Another result of this study was the developed cut-off value for the hsCRP/albumin ratio. A cut-off value for the hsCRP/albumin ratio was calculated to be 0.625 for mortality and 0.500 for recurrence, which may be a reference value for clinical practice. Unlike the CRP/albumin ratio, there is currently a lack of research focusing on the hsCRP/albumin ratio, which presents difficulty in finding other cut-off points for comparison. The cut-off values for the CRP/albumin ratio were reported in 10 solid cancer patients to be in the range of 0.03–0.67 [27]. In addition, the CRP/albumin ratio showed high values of 8.7 and 5.09, respectively, in patients with severe sepsis or septic shock [28,29]. Given that the CRP/albumin ratio also shows various cut-off values depending on cancer type and severity, further studies will be needed to better understand the optimal cut-off value for the hsCRP/albumin ratio in different clinical scenarios.

This study has a few limitations to be considered. First, this study used postoperative hsCRP since it is more sensitive than the usual CRP. However, it cannot be concluded from this retrospective study that hsCRP is superior to CRP in predicting prognosis. Further study is needed on the comparison of hsCRP/albumin ratio and CRP/albumin ratio in terms of predicting long-term prognosis. Second, there could be issues with generalizability since this study was conducted at a single hospital. Third, this investigation was based on medical records accumulated over 10 years from 2004 to 2013, but long-term developments in the medical environment and medical staff may not be reflected. Fourth, postoperative hsCRP/albumin ratio was used instead of the preoperative value in the current study. The preoperative hsCRP/albumin ratio before the operation is useful for selecting the treatment strategy and the operative procedure, whereas postoperative value may be influenced by several parameters including the operation itself, the use of intraoperative albumin or transfusion, and early postoperative complications. Additionally, hsCRP/albumin ratio was measured at POD 0 or 1 without a standardized time point.

In conclusion, the postoperative risk of both mortality and recurrence increased proportionally with the postoperative hsCRP/albumin ratio. In the future, further studies are warranted to validate the hsCRP/albumin ratio as a novel prognostic marker for patients with HCC.

## Figures and Tables

**Table 1:** Baseline characteristics.

Variable		Total (389)	Mean	SD
Sex: male		299 (76.9%)		
Age (year)			58.30	11.73
Body Mass Index (kg m^−2^)			23.86	3.39
Charson Comorbidity Index			3.08	1.17
ASA class	I	103 (26.5%)		
	II	255 (65.6%)		
	III + IV	31 (8.0%)		
Preoperative serum bilirubin (mg dl^−1^)			0.90	0.64
Preoperative aFP (IU mL^−1^)			926.64	4111.91
Preoperative PT INR			1.35	5.07
Preoperative ALT (U L^−1^)			43.37	41.05
Preoperative AST (U L^−1^)			43.50	46.17
Procedures	Laparotomy	232 (59.6%)		
	Laparoscopy	157 (40.4%)		
Operation time (min)			295.54	154.33
Estimated Blood Loss (mL)			1071.31	1844.42
Preoperative TACE		107 (27.5%)		
Preoperative RFA		26 (6.7%)		
Preoperative Child pugh class	A	341 (87.7%)		
	B	35 (9.0%)		
	C	13 (3.3%)		
Virology	HCV (+)	279 (71.7%)		
	HBV (+)	27 (6.9%)		
	Both (+)	1 (0.3%)		
	Both (−)	82 (21.1%)		
Intraoperative Transfusion		106 (27.2%)		
Intraoperative Pringle manuever		73 (18.8%)		
Liver cirrhosis		156 (40.1%)		
Intraoperative Ascites		26 (6.7%)		
Tumor size (cm) *			4.05	2.88
Resection margin (R0)		368 (94.6%)		
Pathologic Tumor stage	1	194 (49.9%)		
	2	141 (36.2%)		
	3	40 (10.3%)		
	4	14 (3.6%)		
Postoperative complication		87 (22.4%)		
Postoperative hsCRP (mg L^−1^)			32.16	41.40
Postoperative albumin (g L^−1^)			38.00	7.76
Postoperative hsCRP/ALB ratio			0.97	1.46
Death		98 (25.2%)		
Recurrence		193 (49.6%)		

Tumor size (cm). * means maximal diameter of largest Hepatocellular carcinoma. SD, Standard Deviation; ASA, American Society of Anesthesiologists; aFP, alpha Feto-Protein; PT INR. Prothrombin time International Normalized Ratio; ALT, alanine aminotransferase; AST, aspartate aminotransferase; TACE, Transarterial chemoembolization; Radiofrequency ablation; HCV, Hepatitis C virus; HBV, Hepatitis B virus; hsCRP, high-sensitivity C-reactive Protein; ALB, Albumin.

**Table 2:** Multivariate Cox regression analysis for mortality after HCC surgery.

Variables		Univariate Analysis		Multivariate Analysis	
		Hazard Ratio (95% CI)	*p*-Value	Hazard Ratio (95% CI)	*p*-Value
Age		1.015 (0.998–1.033)	0.090	1.008 (0.990–1.026)	0.372
Sex: Female (Ref: Male)		0.887 (0.542–1.451)	0.633		
Body Mass Index		0.915 (0.856–0.979)	0.008	0.901 (0.837–0.970)	0.005
ASA class (Ref: I)	II	1.176 (0.743–1.860)	0.489		
	III + IV	1.472 (0.689–3.142)	0.318		
Charson Comorbidity Index score		0.969 (0.805–1.166)	0.738		
Preoperative_TACE		1.051 (0.810–1.365)	0.707		
Preoperative_RFA		1.230 (0.537–2.815)	0.624		
Child_pugh_class B + C (Ref: A)		1.599 (1.125–2.275)	0.009	1.270 (0.794–2.029)	0.319
Preoperative serum bilirubin		0.883 (0.621–1.256)	0.490		
Preoperative PT INR		0.503 (0.082–3.083)	0.457		
Preoperative alanine aminotransferase		1.001 (0.997–1.006)	0.551		
Preoperative aspartate aminotransferase		0.999 (0.994–1.004)	0.759		
Preop Virology	HBV (Ref: HCV)	1.126 (0.670–1.890)	0.655		
	Both Positive (Ref: HCV)	1.082 (0.40–2.601)	0.861		
	Both Negative (Ref: HCV)	3.314 (0.441–24.89)	0.244		
Preoperative serum aFP		1.00 (1.00–1.00)	0.632		
Laparoscopy (Ref: Laparotomy)		2.118 (1.411–3.179)	<0.001	1.270 (0.794–2.029)	0.319
Intraoperative RFA		0.860 (0.398–1.859)	0.701		
Major resection (ref: minor resection) *		1.972 (1.324–2.938)	0.001	1.134 (0.688–1.869)	0.621
Intraoperative ascites		1.671 (0.890–3.138)	0.110		
Intraoperative Pringle manuever		1.068 (0.956–1.194)	0.242		
Operation time (min)		1.001 (0.999–1.002)	0.322		
Estimated Blood Loss (mL)		1.00 (1.00–1.00)	0.095		
Intraoperative Transfusion		0.655 (0.434–0.989)	0.044		
Preoperative Liver Cirrhosis		1.258 (0.822–1.924)	0.291	1.015 (0.612–1.681)	0.955
Pathologic Tumor Size (mm) **		1.119 (1.060–1.182)	<0.001	1.064 (0.993–1.139)	0.079
Tumor number		1.138 (0.843–1.537)	0.398		
Resection margin R1 (Ref: R0)		2.477 (1.244–4.931)	0.010	1.822 (0.790–4.198)	0.159
Pathologic Tumor stage (1 increase in 1–4)		1.448 (1.226–1.709)	<0.001	1.365 (1.134–1.643)	0.001
Postoperative Complication		1.708 (1.119–2.607)	0.013	1.239 (0.743–2.067)	0.411
hsCRP/ALB ratio (continous) ***		1.202 (1.116–1.294)	<0.001	1.171 (1.072–1.278)	<0.001
hsCRP/ALB ratio >0.625 (dichotomous)		2.671 (1.769–4.034)	<0.001	2.257 (1.470–3.466)	<0.001

All covariates of *p* < 0.1 in univariate Cox regression analysis were included in multivariate Cox regression model. HR of hsCRP/ALB ratio (continuous) *** was derived from another Cox proportional hazard model. Major resection * includes resection of four or more liver segments. Tumor size (mm) ** means maximal diameter of largest Hepatocellular carcinoma. HCC, Hepatocellular Carcinoma; Confidence Interval, CI; ASA, American Society of Anesthesiologists; TACE, Transarterial chemoembolization; Radiofrequency ablation; PT INR. Prothrombin time International Normalized Ratio; HCV, Hepatitis C virus; HBV, Hepatitis B virus; aFP, alpha Feto-Protein; hsCRP, high-sensitivity C-reactive Protein; ALB, Albumin.

**Table 3:** Multivariate Cox regression analysis for recurrence after HCC surgery.

Variables		Univariate Analysis		Multivariate Analysis	
		Hazard Ratio (95% CI)	*p*-Value	Hazard Ratio (95% CI)	*p*-Value
Age		0.990 (0.978–1.002)	0.093	0.983 (0.970–0.995)	0.006
Sex: Female (Ref: Male)		0.662 (0.459–0.955)	0.027	0.741 (0.504–1.088)	0.126
Body Mass Index		0.953 (0.911–0.997)	0.036	0.932 (0.887–0.978)	0.004
ASA class (Ref: I)	II	1.240 (0.888–1.732)	0.206		
	III + IV	1.388 (0.775–2.486)	0.270		
Charson Comorbidity Index score		0.99 (0.874–1.121)	0.873		
Preoperative_TACE		0.858 (0.639–1.151)	0.306		
Preoperative_RFA		0.680 (0.418–1.106)	0.120		
Child_pugh_class B + C (Ref: A)		1.185 (0.879–1.597)	0.265		
Preoperative serum bilirubin		1.028 (0.862–1.225)	0.759		
Preoperative PT INR		0.964 (0.844–1.101)	0.587		
Preoperative alanine aminotransferase		1.003 (1.00–1.006)	0.028	1.003 (1.000–1.005)	0.058
Preoperative aspartate aminotransferase		1.001 (0.999–1.003)	0.473		
Preop Virology	HBV (Ref: HCV)	1.015 (0.713–1.445)	0.934		
	Both Positive (Ref: HCV)	1.398 (0.801–2.440)	0.239		
	Both Negative (Ref: HCV)	0.00 (0.00–4.99 × 10^7^)	0.951		
Preoperative serum AFP		1.00 (1.00–1.00)	0.374		
laparoscopy (Ref: laparotomy)		1.291 (0.973–1.714)	0.077	0.970 (0.745–1.433)	0.844
Intraoperative RFA		0.534 (0.328–0.870)	0.012		
Intraoperative ascites		1.364 (0.817–2.276)	0.235		
Major resection (ref: minor resection) *		0.980 (0.719–1.335)	0.897		
Intraoperative Pringle manuever		0.936 (0.780–1.123)	0.478		
Operation time (min)		1.00 (1.00–1.001)	0.362		
Estimated Blood Loss (ml)		1.00 (1.00–1.00)	0.520		
Intraoperative Transfusion		0.800 (0.590–1.084)	0.150		
Preoperative Liver Cirrhosis		1.160 (0.866–1.553)	0.319		
Pathologic Tumor Size (cm) **		1.082 (1.035–1.131)	0.001	1.049 (0.995–1.106)	0.079
Tumor number		1.391 (1.158–1.670)	<0.001	1.126 (1.000–1.478)	0.050
Resection margin R1 (Ref:R0)		2.312 (1.361–3.927)	0.002	2.418 (1.381–4.234)	0.002
Pathologic Tumor stage (1 increase in 1–4)		1.281 (1.118–1.468)	<0.001	1.248 (0.981–1.367)	0.083
Postoperative Complication		1.412 (1.026–1.941)	0.034	1.114 (0.797–1.558)	0.528
hsCRP/ALB ratio (continous) ***		1.210 (1.136–1.289)	<0.001	1.190 (1.108–1.278)	<0.001
hsCRP/ALB ratio > 0.500 (dichotomous)		1.652 (1.240–2.199)	0.001	1.518 (1.125–2.050)	0.006

All covariates of *p* < 0.1 in univariate Cox regression analysis were included in multivariate Cox regression model. Major resection * includes resection of four or more liver segments. Tumor size (mm) ** means maximal diameter of largest Hepatocellular carcinoma. HR of hsCRP/ALB ratio (countinous) *** was derived from another cox proportional hazard model. HCC, Hepatocellular Carcinoma; Confidence Interval, CI; ASA, American Society of Anesthesiologists; TACE, Transarterial chemoembolization; Radiofrequency ablation; PT INR. Prothrombin time International Normalized Ratio; HCV, Hepatitis C virus; HBV, Hepatitis B virus; aFP, alpha Feto-Protein; hsCRP, high-sensitivity C-reactive Protein; ALB, Albumin.

**Table 4:** Cox proportional hazard model for survival or recurrence after HCC surgery according to procedures (laparoscopy and laparotomy).

Variables	Mortality	
	Hazard Ratio (95% CI)	*p*-Value *
Laparoscopy (hsCRP/ALB ratio >0.625)	4.581 (2.445–8.584)	<0.001
Laparotomy (hsCRP/ALB ratio >0.625)	4.646 (2.082–10.366)	<0.001
	Recurrence	
	Hazard ratio (95% CI)	*p*-value *
Laparoscopy (hsCRP/ALB ratio >0.500)	1.483 (1.003–2.193)	0.048
Laparotomy (hsCRP/ALB ratio >0.500)	1.995 (1.307–3.045)	0.001

* All Hazard ratios in Table 4 are derived from four multivariate backward stepwise Cox regression model. HCC, Hepatocellular Carcinoma; CI, Confidence Interval; hsCRP, high-sensitivity C-reactive Protein; ALB, Albumin.

## Supplementary Materials

The following are available online at http://www.mdpi.com/2077-0383/7/6/139/s1, Figure S1: Receiver Operating Characteristics curve for mortality after resection of hepatocellular carcinoma, Figure S2: Receiver Operating Characteristics curve for recurrence after resection of hepatocellular carcinoma, Table S1: Comparison for characteristics between laparoscopy and laparotomy.

## References

[1] White D.L., Kanwal F., Jiao L., El-Serag H.B. (2016). Epidemiology of hepatocellular carcinoma. Hepatocellular Carcinoma.

[2] Dhir M., Melin A.A., Douaiher J., Lin C., Zhen W.K., Hussain S.M., Geschwind J.F., Doyle M.B., Abou-Alfa G.K., Are C. (2016). A review and update of treatment options and controversies in the management of hepatocellular carcinoma. Ann. Surg..

[3] Forner A., Reig M.E., de Lope C.R., Bruix J. (2010). Current strategy for staging and treatment: The BCLC update and future prospects. Semin. Liver Dis..

[4] Kokudo N., Hasegawa K., Akahane M., Igaki H., Izumi N., Ichida T., Uemoto S., Kaneko S., Kawasaki S., Ku Y. (2015). Evidence-based clinical practice guidelines for hepatocellular carcinoma: The japan society of hepatology 2013 update (3rd JSH-HCC guidelines). Hepatol. Res..

[5] Ettorre G.M., Levi Sandri G.B., Colasanti M., Masciana G., de Werra E., Santoro R., Lepiane P., Montalbano M., Antonini M., Vennarecci G. (2017). Liver resection for hepatocellular carcinoma >/=5 cm. Transl. Gastroenterol. Hepatol..

[6] Pamecha V., Sasturkar S.V., Sinha P.K., Mahansaria S.S., Bharathy K.G.S., Kumar S., Rastogi A. (2017). Major liver resection for large and locally advanced hepatocellular carcinoma. Indian J. Surg..

[7] Yang H., Xiong F., Qi R., Liu Z., Lin M., Rui J., Su J., Zhou R. (2010). Laptm4b-35 is a novel prognostic factor of hepatocellular carcinoma. J. Surg. Oncol..

[8] Poon R.T., Fan S.T., Lo C.M., Liu C.L., Wong J. (2002). Long-term survival and pattern of recurrence after resection of small hepatocellular carcinoma in patients with preserved liver function: Implications for a strategy of salvage transplantation. Ann. Surg..

[9] Casadei Gardini A., Carloni S., Scarpi E., Maltoni P., Dorizzi R.M., Passardi A., Frassineti G.L., Cortesi P., Giannini M.B., Marisi G. (2016). Prognostic role of serum concentrations of high-sensitivity c-reactive protein in patients with metastatic colorectal cancer: Results from the itaca trial. Oncotarget.

[10] Ko Y.J., Kwon Y.M., Kim K.H., Choi H.C., Chun S.H., Yoon H.J., Goh E., Cho B., Park M. (2012). High-sensitivity c-reactive protein levels and cancer mortality. Cancer Epidemiol. Biomark. Prev..

[11] Caras R.J., Lustik M.B., Kern S.Q., McMann L.P., Sterbis J.R. (2017). Preoperative albumin is predictive of early postoperative morbidity and mortality in common urologic oncologic surgeries. Clin. Genitourin Cancer.

[12] Lin M.Y., Liu W.Y., Tolan A.M., Aboulian A., Petrie B.A., Stabile B.E. (2011). Preoperative serum albumin but not prealbumin is an excellent predictor of postoperative complications and mortality in patients with gastrointestinal cancer. Am. Surg..

[13] Oh J., Kim S.H., Park K.N., Oh S.H., Kim Y.M., Kim H.J., Youn C.S. (2017). High-sensitivity c-reactive protein/albumin ratio as a predictor of in-hospital mortality in older adults admitted to the emergency department. Clin. Exp. Emerg. Med..

[14] Yang C., Yang Y., Li B., Xu P., Shen Q., Yang Q. (2016). The diagnostic value of high-sensitivity c-reactive protein/albumin ratio in evaluating early-onset infection in premature. Zhonghua Wei Zhong Bing Ji Jiu Yi Xue.

[15] Ma L.N., Liu X.Y., Lu Z.H., Wu L.G., Tang Y.Y., Luo X., Hu Y.C., Yan T.T., Wang Q., Ding X.C. (2017). Assessment of high-sensitivity c-reactive protein tests for the diagnosis of hepatocellular carcinoma in patients with hepatitis b-associated liver cirrhosis. Oncol. Lett..

[16] Nault J.C., Guyot E., Laguillier C., Chevret S., Ganne-Carrie N., N’Kontchou G., Beaugrand M., Seror O., Trinchet J.C., Coelho J. (2013). Serum proteoglycans as prognostic biomarkers of hepatocellular carcinoma in patients with alcoholic cirrhosis. Cancer Epidemiol. Biomark. Prev..

[17] Tateishi R., Shiina S., Yoshida H., Teratani T., Obi S., Yamashiki N., Yoshida H., Akamatsu M., Kawabe T., Omata M. (2006). Prediction of recurrence of hepatocellular carcinoma after curative ablation using three tumor markers. Hepatology.

[18] Fujiwara N., Tateishi R., Nakagawa H., Nakagomi R., Kondo M., Minami T., Sato M., Uchino K., Enooku K., Kondo Y. (2015). Slight elevation of high-sensitivity c-reactive protein to predict recurrence and survival in patients with early stage hepatitis c-related hepatocellular carcinoma. Hepatol. Res..

[19] Liu Y.B., Ying J., Kuang S.J., Jin H.S., Yin Z., Chang L., Yang H., Ou Y.L., Zheng J.H., Zhang W.D. (2015). Elevated preoperative serum hs-crp level as a prognostic factor in patients who underwent resection for hepatocellular carcinoma. Medicine (Baltimore).

[20] Kinoshita A., Onoda H., Imai N., Iwaku A., Oishi M., Tanaka K., Fushiya N., Koike K., Nishino H., Matsushima M. (2015). The c-reactive protein/albumin ratio, a novel inflammation-based prognostic score, predicts outcomes in patients with hepatocellular carcinoma. Ann. Surg. Oncol..

[21] Han H.S., Shehta A., Ahn S., Yoon Y.S., Cho J.Y., Choi Y. (2015). Laparoscopic versus open liver resection for hepatocellular carcinoma: Case-matched study with propensity score matching. J. Hepatol..

[22] Yoon Y.S., Han H.S., Cho J.Y., Ahn K.S. (2010). Total laparoscopic liver resection for hepatocellular carcinoma located in all segments of the liver. Surg. Endosc..

[23] Goh B.K., Teo J.Y., Chan C.Y., Lee S.Y., Jeyaraj P., Cheow P.C., Chow P.K., Ooi L.L., Chung A.Y. (2016). Importance of tumor size as a prognostic factor after partial liver resection for solitary hepatocellular carcinoma: Implications on the current ajcc staging system. J. Surg. Oncol..

[24] Pang R.W., Poon R.T. (2012). Diagnosis: Novel prognostic biomarkers in hepatocellular carcinoma. Nat. Rev. Gastroenterol. Hepatol..

[25] Gabay C., Kushner I. (1999). Acute-phase proteins and other systemic responses to inflammation. N. Engl. J. Med..

[26] Hubner M., Mantziari S., Demartines N., Pralong F., Coti-Bertrand P., Schafer M. (2016). Postoperative albumin drop is a marker for surgical stress and a predictor for clinical outcome: A pilot study. Gastroenterol. Res. Pract..

[27] Li N., Tian G.W., Wang Y., Zhang H., Wang Z.H., Li G. (2017). Prognostic role of the pretreatment c-reactive protein/albumin ratio in solid cancers: A meta-analysis. Sci. Rep..

[28] Kim M.H., Ahn J.Y., Song J.E., Choi H., Ann H.W., Kim J.K., Kim J.H., Jeon Y.D., Kim S.B., Jeong S.J. (2015). The c-reactive protein/albumin ratio as an independent predictor of mortality in patients with severe sepsis or septic shock treated with early goal-directed therapy. PLoS ONE.

[29] Ranzani O.T., Zampieri F.G., Forte D.N., Azevedo L.C., Park M. (2013). C-reactive protein/albumin ratio predicts 90-day mortality of septic patients. PLoS ONE.

