# Supplementary material for: The High-Sensitivity C-Reactive Protein/Albumin Ratio Predicts Long-Term Oncologic Outcomes after Curative Resection for Hepatocellular Carcinoma
Source: J Clin Med. 2018 Jun 7;7(6):139. doi: 10.3390/jcm7060139 (PMC6024983; doi:10.3390/jcm7060139)
Supplement: Supplementary file 1 [file jcm-07-00139-s001.pdf]

Supplemental Material: The High-Sensitivity C-Reactive Protein/Albumin Ratio Predicts Long-Term Oncologic Outcomes after Curative Resection for Hepatocellular Carcinoma

Table S1. Comparison for characteristics between laparoscopy and laparotomy.

Variables	Laparoscopy	Laparotomy	*P*-value
	N = 232	*N* = 157	
Age (year)	57.38 (11.52)	59.66 (11.93)	0.059
Sex: Male	165 (71.1%)	134 (85.4%)	0.001
Body Mass Index (kg m^{-2})	24.18 (3.69)	23.36 (2.89)	0.020
Charson Comorbidity Index	3.16 (1.17)	2.97 (1.15)	0.134
Preoperative ASA class			0.355
I	60 (25.9%)	43 (27.4%)	
II	157 (67.7%)	98 (62.4%)	
III, IV	15 (6.4%)	16 (10.2%)	
Preoperative Child Pugh Class			0.016
A	212 (91.4%)	129 (82.2%)	
B	16 (6.9%)	19 (12.1%)	
C	4 (1.7%)	9 (5.7%)	
Preoperative TACE	54 (23.3%)	53 (33.8%)	0.033
Preoperative RFA	16 (6.9%)	10 (6.4%)	0.838
Operation time (min)	287.99 (174.70)	117.62 (9.39)	0.241
Estimated Blood Loss (ml)	1043.10 (2068.37)	1112.99 (1457.11)	0.714
Length of hospital stay (day)	9.84 (10.76)	16.22 (18.37)	<0.001
Tumor number	1.21 (0.49)	1.32 (0.71)	0.054
Intraoperative Pringle manuever	31 (13.4%)	42 (26.8%)	0.004
Resection margin: R0	225 (97.0%)	143 (91.1%)	0.012
Pathologic Tumor stage			<0.001
1	127 (54.7%)	67 (42.7%)	
2	89 (38.4%)	52 (33.1%)	
3	9 (3.9%)	31 (19.75)	
4,5	7 (3.1%)	7 (4.4%)	
Type of Resection: Major*	195 (84.1%)	76 (48.4%)	<0.001
Pathologic Tumor Size (cm)**	3.11 (1.74)	4.94 (2.85)	<0.001
Postoperative complication	38 (16.4%)	49 (31.2%)	0.001
Postoperative hsCRP/ALB	0.87 (1.16)	1.06 (1.86)	0.208

Values are expressed as the mean (standard deviation) or number (percentage, %). Major resection* includes resection of four or more liver segments; Tumor size (mm)** means maximal diameter of largest Hepatocellular carcinoma. SD, Standard Deviation; ASA, American society of anesthesiologists; TACE, Transarterial chemoembolization; Radiofrequency ablation; hsCRP, high sensitivity C-reactive Protein; ALB, Albumin

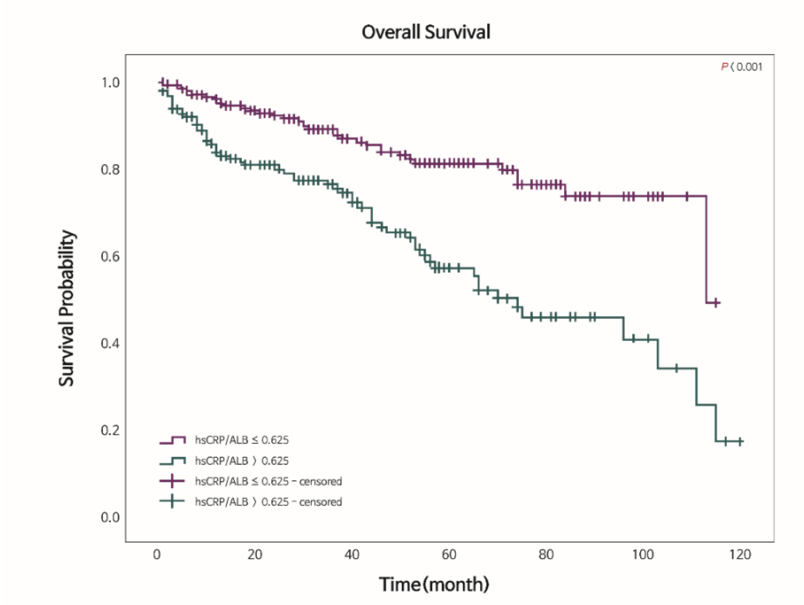

Figure S1. Receiver Operating Characteristics curve for mortality after resection of hepatocellular carcinoma. Area under curve from receiver operating characteristic analysis: 0.692; 95% confidence interval: 0.635–0.748.

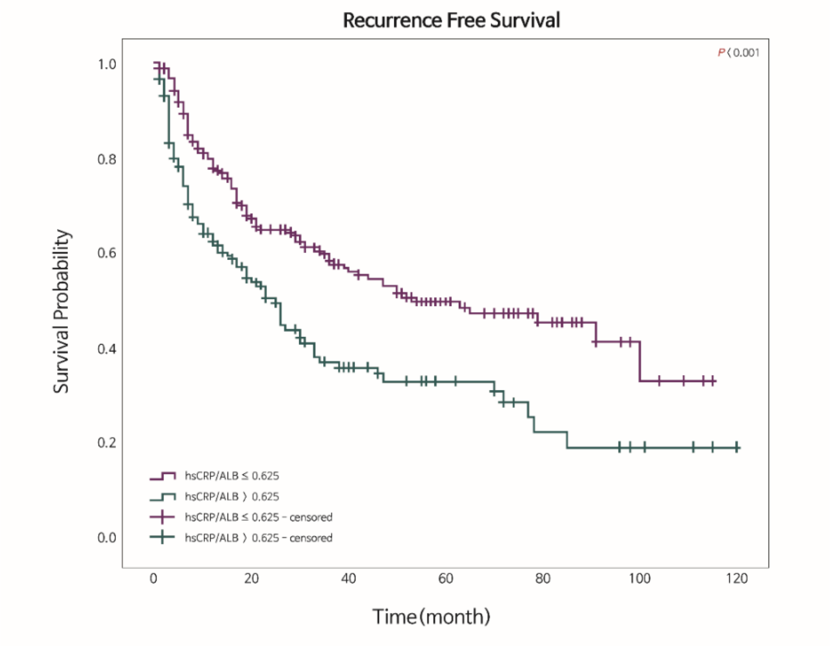

Figure S2. Receiver Operating Characteristics curve for recurrence after resection of hepatocellular carcinoma. Area under curve from receiver operating characteristic analysis: 0.587; 95% confidence interval: 0.536–0.637.
